# Rare Presentation of Testicular Cancer With Malignant Tumor Thrombus: A Case Report

**DOI:** 10.7759/cureus.69857

**Published:** 2024-09-21

**Authors:** Abdulaziz H Khushaym, Noora Aljeeran, Lana Alabbasi

**Affiliations:** 1 Urology, King Hamad University Hospital, Busaiteen, BHR; 2 Internal Medicine, King Hamad University Hospital, Busaiteen, BHR

**Keywords:** ivc tumor thrombus in testicular cancer, malignant tumor thrombus, management of testicular cancer with vascular complications, metastatic testicular cancer, neoplasmatic thrombus in ivc

## Abstract

In rare cases, testicular cancer can metastasize to the inferior vena cava (IVC) through a tumor thrombus, significantly worsening the prognosis. This case report describes the management and the outcome of a rare presentation of testicular cancer. A 38-year-old male presented with a testicular mass and was found to have a mixed germ cell tumor with predominantly yolk sac components. Imaging after the initial orchiectomy revealed an IVC tumor thrombus extending from the left renal vein, as well as retroperitoneal and pulmonary metastases. The patient was urgently started on anticoagulation and underwent three cycles of BEP chemotherapy (bleomycin, etoposide, and cisplatin), which led to complete metabolic and radiographic regression of the metastatic disease.

## Introduction

Testicular cancer is the most prevalent type of solid tumor affecting men under 45 years old [1]. Testicular cancer can be classified into several histological categories: sex cord-stromal tumors, germ cell tumors, and mixed types. Germ cell tumors, which account for approximately 95% of testicular cancer cases, are the most prevalent. Within germ cell tumors, there are two main subtypes: seminomatous and nonseminomatous [1]. Nonseminomatous subtypes typically grow rapidly and have the capacity to invade the bloodstream, which raises their risk of distant metastasis [2]. Possible common sites for testicular cancer metastasis include the adjacent lymph nodes; additionally, distant metastases can occur in the lungs and the abdominal organs [3].

An uncommon type of testicular cancer metastasis is tumor thrombus, which refers to intravascular tumor invasion, particularly the inferior vena cava (IVC) [2]. This can be due to various etiologies such as inflammation, prior surgery, prolonged bed rest, and deep vein thrombosis in the lower limbs. The thrombus in the IVC may either be cancerous (neoplastic) or non-cancerous (non-neoplastic) in nature. Imaging techniques like CT scans and color Doppler ultrasound play an important role in determining the character and composition of the IVC thrombus [4]. There are two mechanisms through which IVC is involved. The first cause is due to the direct spread of the tumor through invasion of the spermatic vein and then the vena cava. Thus, right-sided tumors are more frequently seen in IVC invasion due to the involvement of the right gonadal vein. The second cause would be through direct lymphatic spread by invading the paracaval metastatic sites through developing lymphatic-venous shunting in severe lymphatic disease [5].

The most significant complication of IVC thrombosis is pulmonary embolism leading to death. Moreover, the extension of tumoral thrombus particularly to the heart increases the risk of embolism [5]. It is estimated that IVC involvement occurs in somewhere between 3% and 11% of testicular cancer cases. This complication significantly worsens the prognosis and impacts the treatment approach for these patients [3].

## Case presentation

**Figure 1:**
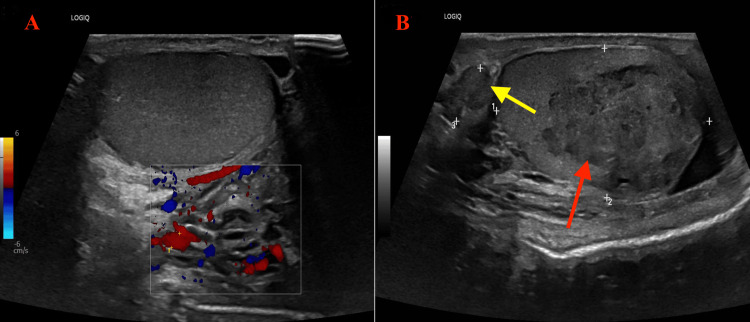
Scrotal ultrasound with color Doppler A: Normal right testis with normal vascularity. B: Heterogeneous and ill-defined mass lesion in the left testis, occupying the mid and lower poles (red arrow).

A 38-year-old male with no significant medical history presented at a urology clinic with mild-moderate left flank pain radiating to the groin and testis for three months. He reported no history of dysuria, urinary frequency, nocturia, hematuria, penile discharge, or trauma. On physical exam, his left epididymis was thickened and tender, with thickness toward the lower pole testis; otherwise, the examination was unremarkable. A scrotal ultrasound with color Doppler (Figure 1) was performed and showed a heterogenous and ill-defined mass lesion in the left testis of 3.3 x 2.1 cm occupying the mid and lower poles. The mass was associated with a few small areas of calcification within this area, distorting the testicular parenchyma. The vascularity in the remaining upper pole was unremarkable. It was also associated with a small hydrocele. The right testis is normal in contour and size, measuring 4.8 x 2.1 cm. No other lesion was seen in the right testis, and the right epididymis is unremarkable (Figure 1). Additional laboratory testing was ordered at that visit, including alpha-fetoprotein (AFP), quantitative beta-human chorionic gonadotropin (hCG), and lactate dehydrogenase (LDH) (Table 1).

**Table 1:** Initial laboratory investigations

Parameters	Findings	Reference ranges
Alpha-fetoprotein	8.90 lu/ml	0-40 lu/ml
Beta-human chorionic gonadotropin	86.8 mlu/ml	0.02-0.8 mlu/ml
Lactate dehydrogenase	362 IU/L	140-280 IU/L

A CT scan with contrast of the chest, abdomen, and pelvis was ordered to rule out any metastasis and was unremarkable, except for enlarged retroperitoneal nodes involving the left paraaortic nodes and the paracaval nodes. The larger nodes measure about 2 cm in the short axis, likely representing metastatic nodal deposits. The patient underwent a left radical orchidectomy through an inguinal approach. The hospital stay was uneventful, and he was then discharged home in stable condition on the second day after the surgery on Eliquis (apixaban) 5 mg twice daily with a follow-up appointment the week after for a laboratory (Table 2) and histopathology investigations, which revealed a mixed germ cell tumor with a predominantly yolk sac tumor component (about 80% of all tumor cells), with a tumor size of 4.1 cm. The tumor invaded the spermatic cord, blood vessels, epididymis, and tunica albuginea (TNM stage: pT3N1M0).

**Table 2:** Laboratory investigations  post left radical orchidectomy

Parameter	Finding	Reference Range
Alpha-fetoprotein	24.9 lu/ml	0-40 lu/ml
Beta-human chorionic gonadotropin	59.8 mlu/ml	0.02-0.8 mlu/ml
Lactate dehydrogenase	553 IU/L	140-280 IU/L

The patient's case was presented at a national tumor board, and postoperative restaging with F-18-FDG PET-CT scans was planned. However, after the PET-CT was performed, the radiologist contacted the responsible physician with a concerning finding from the CT scan.

**Figure 2:**
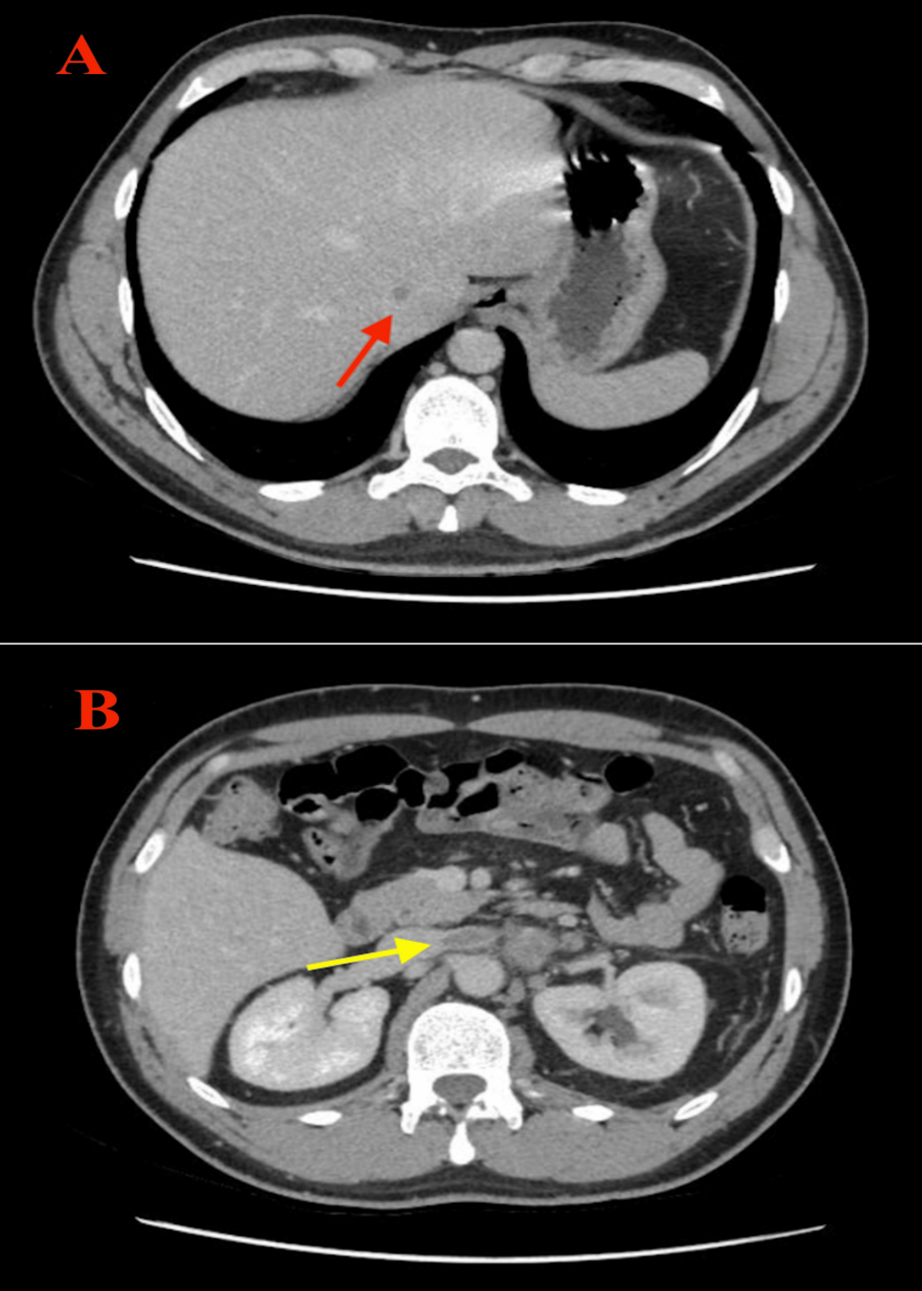
CT scan of abdomen A: The defect from the beginning of the vena cava (red arrow). B: The extension to the left renal vein (yellow arrow).

The CT scan (Figure 2) revealed a linear, intravascular fluorodeoxyglucose (FDG) uptake tracking along the left renal vein and extending into the hepatic portion of the inferior vena cava, suggesting the presence of a malignant tumor thrombus. There was no FDG-avid recurrence or residual disease at the operative site. The PET-CT did, however, demonstrate multiple, high-grade, hypermetabolic abdominal and pelvic retroperitoneal nodal metastases, as well as pulmonary metastases. The rest of the body showed no signs of abnormal FDG uptake.

The patient was then urgently called to come to the emergency department. He was also started on venous thromboembolism (VTE) prophylaxis with anticoagulation. Before beginning treatment, a coagulation profile was ordered, which was normal (Table 3).

**Table 3:** Coagulation profile result prior to starting venous thromboembolism prophylaxis

Parameter	Finding	Reference Range
Prothrombin Time	11.3 sec	10.5-13.5 sec
APTT	27.1 sec	25-35 sec
APTT Ratio	1.04%	0.9 -1.4%
INR	0.96	0.90-1.12

**Figure 3:**
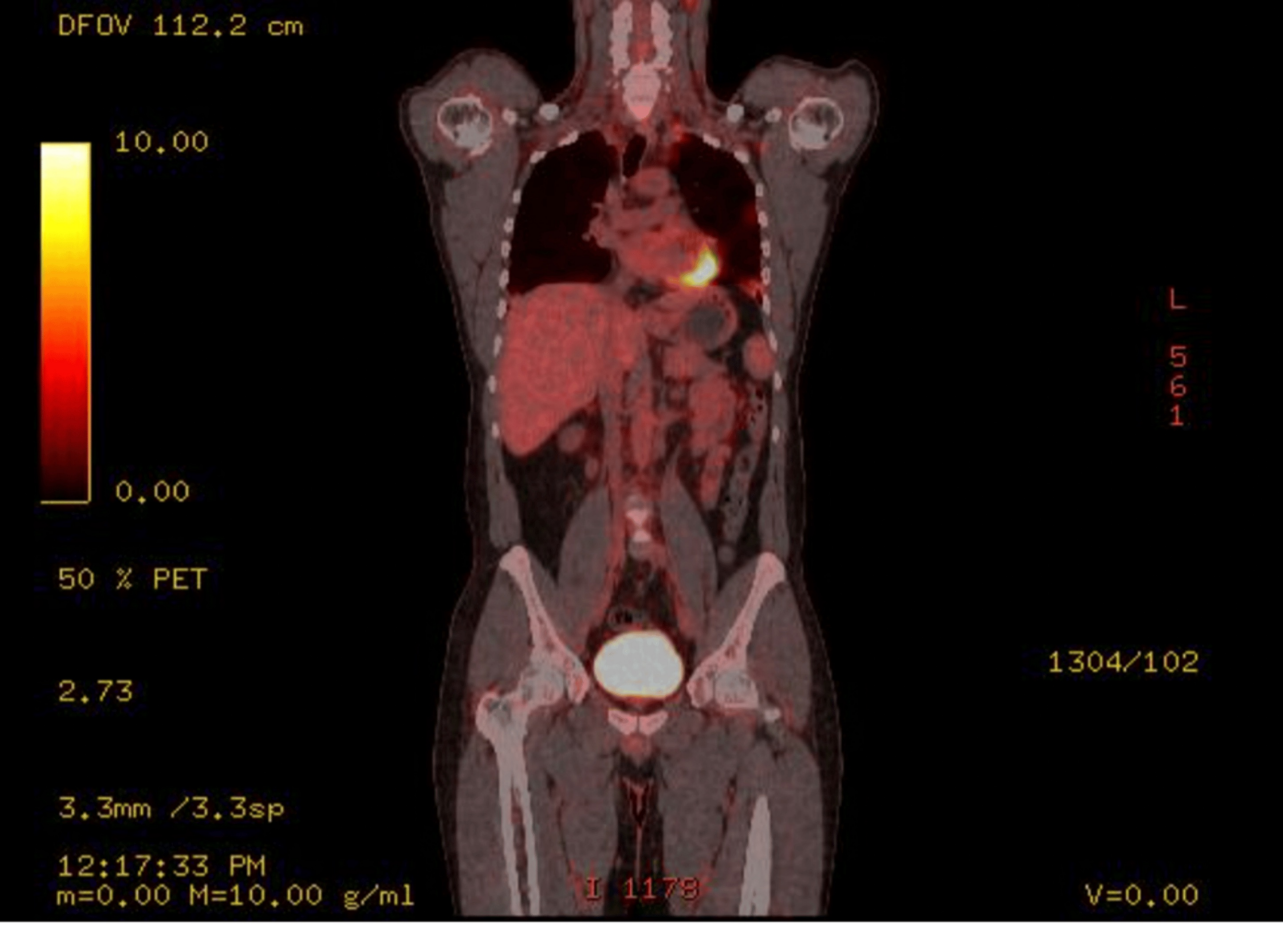
PET-CT post chemotherapy showing complete regression of the disease

The patient was then started on a course of chemotherapy. He received three cycles of BEP (bleomycin, etoposide, and cisplatin) chemotherapy, administered once every three weeks. After completion of the chemotherapy regimen, a follow-up PET-CT scan was performed (Figure 3), and a hormonal laboratory investigation was also conducted (Table 4). The imaging showed complete metabolic regression of the previously seen multiple enlarged lymph nodes in the left iliac, left para-aortic, and aorto-caval regions, with no newly developed FDG-avid abdominal or pelvic nodal lesions detected. There was resolution of the previously noted left ureteric compression caused by the nodal deposits. The PET-CT also demonstrated complete metabolic regression of the previously seen hypermetabolic malignant thrombus involving the left renal vein and extending into the hepatic portion of the inferior vena cava, displaying a complete resolution of the previously seen hypermetabolic pulmonary nodules.

**Table 4:** Laboratory investigations post-chemotherapy

Parameter	Finding	Reference Range
Alpha-fetoprotein(AFP)	5.1 lu/ml	0-40 lu/ml
beta-human chorionic gonadotropin (hCG)	1 mlu/ml	0.02-0.8 mlu/ml
lactate dehydrogenase (LDH)	137 IU/L	140-280 IU/L

The patient's case was discussed at a national tumor board. The recommendations were to conduct close surveillance, with PET-CT imaging at two months, repeat laboratory tests, and a continuation of anticoagulation with Eliquis 5 mg twice daily for at least six months (due to the malignancy being the underlying reason for the thrombus). Further PET-CT review will be scheduled prior to discontinuation of anticoagulation, as this proactive approach aims to monitor for disease progression while appropriately managing the thromboembolic risk. There was no indication of retroperitoneal lymph node dissection at this time.

## Discussion

We report a rare case of metastatic malignant testicular cancer with invasion of the IVC. To diagnose testicular cancer, an orchidectomy must be performed. However, ultrasound imaging is preferred for the initial assessment and evaluation of any suspicious testicular masses or abnormalities [6].

On ultrasound imaging, the typical testicular tumor will appear as a solid mass within the testicular tissue that is hypoechoic or has a heterogeneous echogenic pattern. However, some malignant testicular tumors may also demonstrate cystic regions or areas of necrosis within the mass [6]. The diagnosis of a tumor thrombus is primarily determined through a CT scan with contrast or MRI imaging, along with pathological examination of the primary tumor. In these imaging studies, the endoluminal tumor thrombus usually shows similar varied contrast enhancement patterns as the primary tumor [6]. Thus, CT can be helpful in avoiding unnecessary long-term anticoagulation therapy by differentiating non-hypermetabolic thrombosis from hypermetabolic tumoral thrombosis [5].

Complications associated with tumor thrombi can include pulmonary embolism, Budd-Chiari syndrome, cardiac dysfunction, and the formation of varicoceles [7]. A tumor thrombus can also cause complete obstruction or blockage of the blood vessel or may invade through the vessel wall. This can cause expansion of the affected vessel and increase the risk of rupture, potentially resulting in a life-threatening hemorrhage [3]. Early detection and surgical removal of the tumor are important for a positive outcome and for the prevention of tumor thrombus complications [7]. In this case, the contrast-enhanced CT scan was adequate to reveal the malignant invasion and to where it was extended, and there was no need for an MRI study. It provides adequate information about the characteristics of the tumor thrombus and is considered a useful technique for both the interpretation and staging of malignancies [8].

The prognosis for a patient with testicular cancer can be predicted based on multiple factors, including cancer sensitivity to the chemotherapy, the absence of metastases to the brain, liver, or bone, serum levels of AFP being lower than 1000 ng/mL, beta-hCG being either above or below 5000 mIU/mL, and LDH being either above or below three times the upper limit of normal levels. Given these favorable prognostic indicators, the prognosis for the described patient in the case was considered favorable [9].

There are several approaches to treating testicular cancer when a tumor thrombus is present, including chemotherapy alone or combined with surgical removal of the tumor, surgery of the primary tumor without chemotherapy, or the placement of an IVC filter before surgically removing the tumor thrombus. The specific treatment plan will depend on the individual patient's circumstances and the extent of the tumor thrombus involvement [10].

There is a potential link between the use of cisplatin-based chemotherapy regimens and an increased risk of developing VTE in patients with metastatic germ cell tumors [11]. The potential link between cisplatin-based chemotherapy and VTE in patients with metastatic germ cell tumors has been investigated. A retrospective analysis revealed that 19% of patients with testicular germ cell cancer undergoing cisplatin-based chemotherapy experienced VTE, which notably decreased their overall survival [12]. Another retrospective analysis of patients with metastatic germ cell tumors receiving first-line chemotherapy found that the highest risk of thromboembolic events was observed before and during the chemotherapy period, with no significant increase in risk afterward [13]. Strong evidence also supports the use of low-molecular-weight heparin as prophylactic anticoagulation to lower the risk of VTE. Prophylactic anticoagulation has been shown to effectively reduce VTE risk in patients when the risk-benefit profile is favorable. Furthermore, the tumor-induced hypercoagulable state has also been investigated as a possible factor in the formation of tumor thrombus formation [13].

The patient in our case underwent a radical left orchiectomy to treat the primary tumor, and the standard triple-chemotherapy regimen of BEP was used rather than just a dual regimen of etoposide and cisplatin. The decision was made to be aggressive with the chemotherapy, even though the addition of bleomycin increases the risk of interstitial pulmonary fibrosis. The team weighed the risks and benefits and proceeded with the more intensive BEP chemotherapy to effectively treat the tumor thrombus. Additionally, despite the risk of thrombocytopenia associated with heparin therapy, full-dose heparinization was safely used to prevent pulmonary embolism during intensive chemotherapy [9].

Due to the high risk of thrombocytopenia that can occur during heparin therapy, some authors have advocated for surgically removing the vena cava thrombus before administering chemotherapy to prevent potential embolic complications that could arise from the treatment [14]. Others have recommended excising the thrombosed vena cava after completing chemotherapy. This approach is based on the concern that any residual tumor thrombus left in the vena cava could undergo malignant transformation, and thus it is safer to remove it after the chemotherapy has been administered [15]. We felt that the effective chemotherapy regimens available for treating testicular carcinoma would be able to effectively manage the tumor thrombi. Using heparin therapy helps prevent the formation of emboli from any blood clots present in the vena cava. This combination of aggressive chemotherapy and anticoagulation was the preferred approach over surgical removal of the thrombus.

## Conclusions

This case highlights the rare and challenging presentation of testicular cancer and to the best of our knowledge, this is the first reported case of its kind from the Middle East, providing important clinical insights into the diagnosis and management of advanced testicular cancer with vascular complications. It also underscored the role of the radiologist in the early detection of the tumor thrombus, which allowed for prompt initiation of tumor thrombus management. This multidisciplinary approach was essential in achieving a favorable outcome in this rare and high-risk presentation of testicular cancer. Radiologists should maintain a high index of suspicion for IVC tumor thrombus in testicular cancer patients, as early diagnosis can significantly impact management and prognosis. The successful management of this uncommon case demonstrates that with appropriate diagnostic evaluation and a tailored, aggressive therapeutic plan, favorable results can be achieved in high-risk testicular cancer patients, despite the rarity of the presentation.

## References

[1] Quencer KB, Friedman T, Sheth R, Oklu R (2017). Tumor thrombus: incidence, imaging, prognosis and treatment. Cardiovasc Diagn Ther.

[2] Zarour CC, Zaki-Metias KM, Gri J, Cavender J, Stepanek KA, Cotant MB, Allen LS (2021). Testicular cancer with extensive gonadal and renal vein tumor thrombus. Clin Imaging.

[3] Bredael JJ, Vugrin D, Whitmore WF Jr (1982). Autopsy findings in 154 patients with germ cell tumors of the testis. Cancer.

[4] Al-Mondhiry H (1984). Tumor interaction with hemostasis: the rationale for the use of platelet inhibitors and anticoagulants in the treatment of cancer. Am J Hematol.

[5] Dusaud M, Bayoud Y, Desfemmes FR, Molimard B, Durand X (2015). Unusual presentation of testicular cancer with tumor thrombus extending to the inferior vena cava. Case Rep Urol.

[6] Coursey Moreno C, Small WC, Camacho JC (2015). Testicular tumors: what radiologists need to know--differential diagnosis, staging, and management. Radiographics.

[7] Karaosmanoglu AD, Onur MR, Uysal A, Akata D, Ozmen MN, Karcaaltincaba M (2020). Tumor in the veins: an abdominal perspective with an emphasis on CT and MR imaging. Insights Imaging.

[8] Hamm B (1997). Differential diagnosis of scrotal masses by ultrasound. Eur Radiol.

[9] Fein DE, Paulus JK, Mathew P (2018). Reassessment of 4-cycle etoposide and cisplatin as the standard of care for good-risk metastatic germ cell tumors. JAMA Oncol.

[10] Masui S, Onishi T, Arima K, Sugimura Y (2005). Successful management of inferior vena cava thrombus complicating advanced germ cell testicular tumor with temporary inferior vena cava filter. Int J Urol.

[11] Solari L, Krönig M, Ihorst G (2016). High rates of thromboembolic events in patients with germ cell cancer undergoing cisplatin-based polychemotherapy. Urol Int.

[12] Paffenholz P, Grein K, Heidegger I (2019). Predictors of thrombosis in testicular cancer during platinum-based chemotherapy. World J Urol.

[13] Sharifi R, Ray P, Schade SG, Lee M (1988). Inferior vena cava thrombosis. Unusual presentation of testicular tumor. Urology.

[14] Jacqmin D, Bertrand P, Ansieau JP, Dufour P, Bollack C (1989). Involvement of the caval vein lumen by a metastasis of a non-seminomatous testicular tumor. Eur Urol.

[15] Morgentaler A, Garnick MB, Richie JP (1988). Metastatic testicular teratoma invading the inferior vena cava. J Urol.

